# Supplementary material for: Daphne kiusiana Crude Extract and Its Fraction Enhance Keratinocyte Migration via the ERK/MMP9 Pathway
Source: Curr Issues Mol Biol. 2025 Apr 25;47(5):300. doi: 10.3390/cimb47050300 (PMC12110485; doi:10.3390/cimb47050300)
Supplement: Supplementary file 1 [file cimb-47-00300-s001.zip › cimb-3569056-supplementary.pdf]

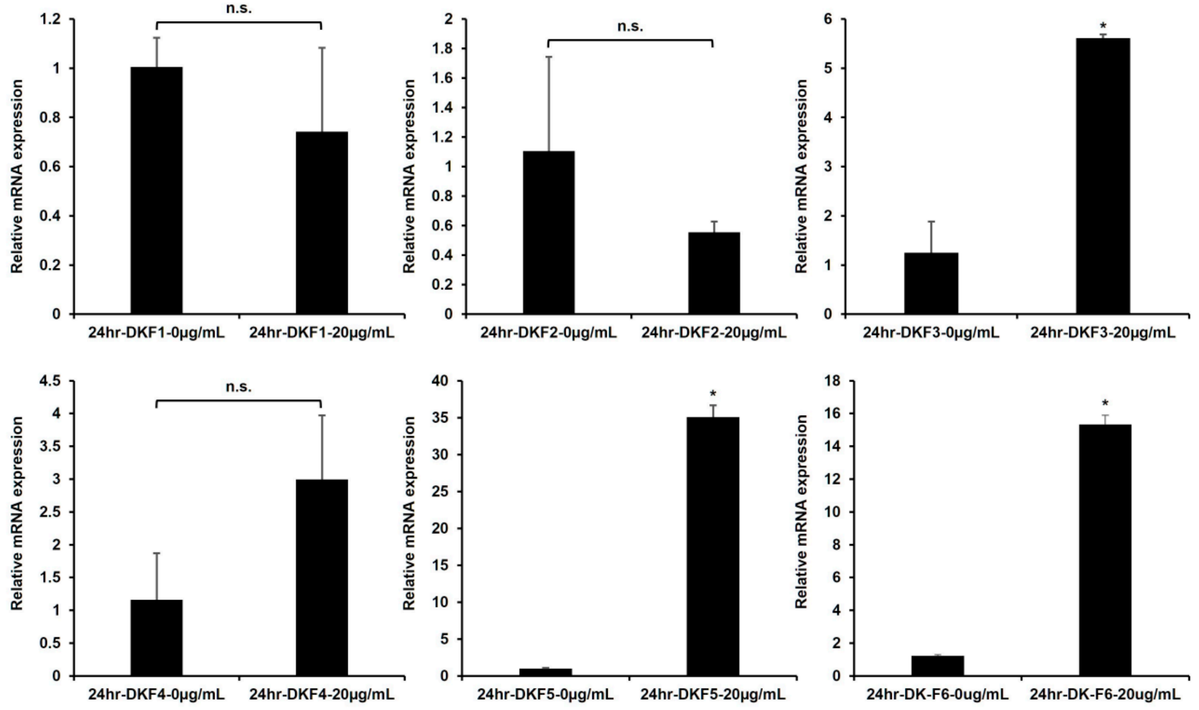

Supplementary Figure S1. The effects of six different fractions from the stem extract of *Daphne kiusiana* (DKS) on MMP9 mRNA expression were evaluated. HaCaT cells were treated with the indicated fractions of *Daphne kiusiana* stem extracts (DKS) for 24 hours, and the expression level of MMP9 was quantified using qRT-PCR, with GAPDH serving as the reference gene. The presented data represents the mean ± SD (n = 3). Statistical significance is denoted by *$p < .05$ compared to 0 μg/mL. The data was analyzed through a paired student *t*-test.

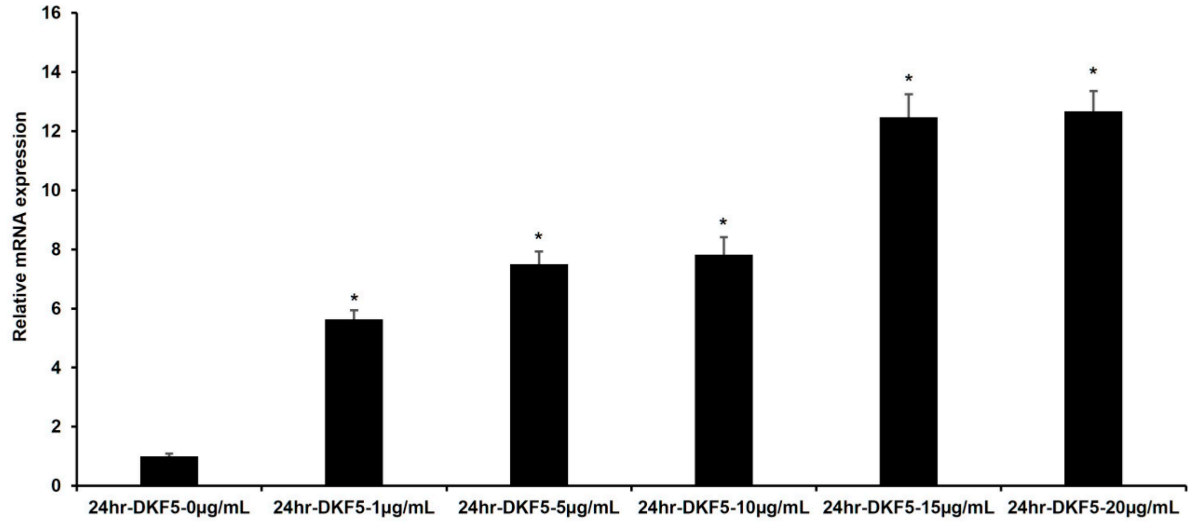

Supplementary Figure S2. Fraction No.5 of stem extract from *Daphne kiusiana* (DKF5) induces MMP9 gene expression in a dose-dependent manner. HaCaT cells were cultured in the presence of fraction No.5 of stem extract from *Daphne kiusiana* (DKF5) at varying concentrations (0–20 μg/mL) for 24 hours. MMP9 gene expression was assessed via qRT-PCR, with GAPDH as the reference gene. The presented data represents the mean ± SD (n = 3). Statistical significance is denoted by *$p < .05$ compared to 0 μg/mL. The data was analyzed through a paired student *t*-test.

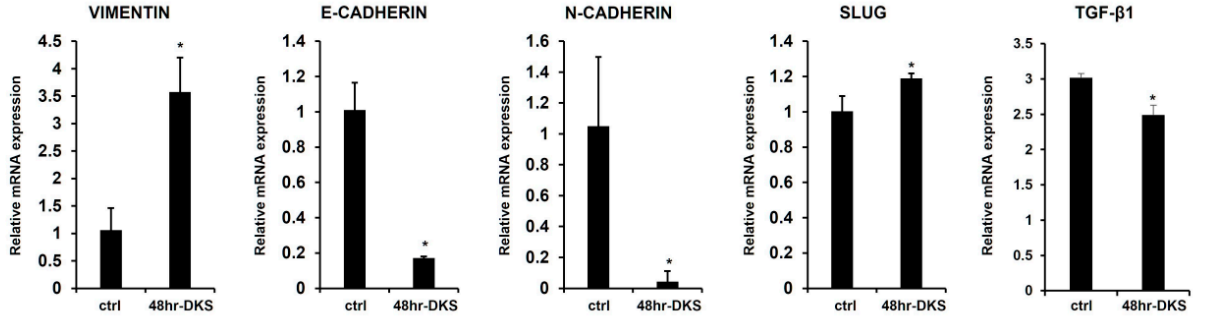

Supplementary Figure S3. The expression pattern of EMT marker genes upon stem extract from *Daphne kiusiana* (DKS) treatment did not follow the typical EMT-related gene regulation observed during keratinocyte migration. HaCaT cells were treated with DKS at 40 μg/mL for 24 hours, followed by analysis of E-cadherin, N-Cadherin, Vimentin, Slug, and TGF-β1 gene expression using qRT-PCR, with GAPDH as the reference gene. The presented data represent the mean ± SD (n = 3). Statistical significance is denoted by *$p < .05$ compared to control. The data was analyzed through a paired student *t*-test.

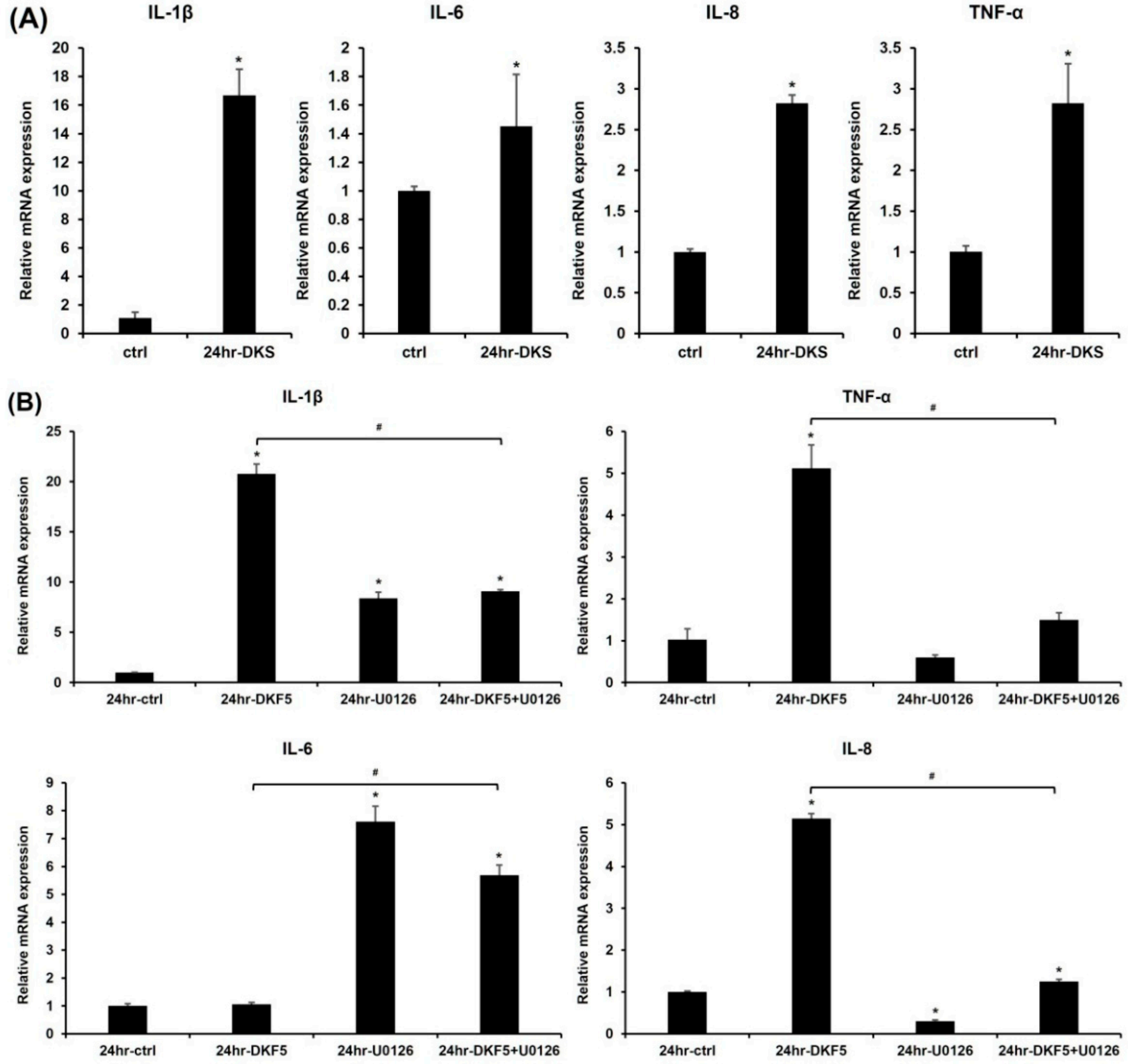

Supplementary Figure S4. *Daphne kiusiana* stem extracts (DKS) and fraction No.5 of stem extract from *Daphne kiusiana* (DKF5) upregulate inflammatory cytokines, and U0126 abrogates fraction No.5 of stem extract from *Daphne kiusiana* (DKF5)-induced effects. (**A**) HaCaT cells were incubated with *Daphne kiusiana* stem extracts (DKS)

at 40 µg/mL for 24 hours. (**B**) In the separate experiment, HaCaT cells were treated with fraction No.5 of stem extract from *Daphne kiusiana* (DKF5) at 1 µg/mL in the presence or absence of selective MEK 1/2 inhibitor U0126 at 10 µM for 24 hours. The expression levels of the indicated inflammatory cytokine genes were analyzed using qRT-PCR. The presented data represents the mean ± SD (n = 3). Statistical significance is denoted by *p < .05 compared to control and #p < .05 compared to fraction No.5 of stem extract from *Daphne kiusiana* (DKF5) treated group. Supplementary Figure S4-A was analyzed through a paired student *t*-test, while Supplementary Figure S4-B was analyzed through One-way ANOVA with Tucky post hoc analysis.

Supplementary Table S1. Human primer sequences used for qRT-PCR in the Supplementary Figures

Gene	Primer sequence
E-CADHERIN	Forward: 5′-CGG AAT GCA GTT GAT C-3′<br>Reverse: 5′-AGG ATG GTG TAA GCG ATC GC-3′
IL-1β	Forward: 5′-CTC CAG GGA CAG GAT ATG GA-3′<br>Reverse: 5′-TCT TTC AAC ACG CAG GAC AG-3′
IL-6	Forward: 5′-ACT CAC CTC TTC AGA ACG AAT TG-3′<br>Reverse: 5′-CCA TCT TTG GAA GGT TCA GGT TG-3′
IL-8	Forward: 5′-TTG GCA GCC TTC CTG ATT TC-3′<br>Reverse: 5′-TAT GCA CTG ACA TCT AAG TTC TTT AGC A-3′
N-CADHERIN	Forward: 5′-CTC CAT GTG CCG GAT AGC-3′<br>Reverse: 5′-CGA TTT CAC CAG AAG CCT CTA C-3′
SLUG	Forward: 5′-TGG GCA AAG AAC TAC TGC G-3′<br>Reverse: 5′-AGAGTTGGCGGAGCTAAACAG-3′
TGF-β1	Forward: 5′-CTA ATG GTG GAA ACC CAC AAC G-3′<br>Reverse: 5′-TAT CGC CAG GAA TTG TTG CTG-3′
TNF-α	Forward: 5′-GAG GCC AAG CCC TGG TAT G-3′<br>Reverse: 5′-CGG GCC GAT TGA TCT CAG C-3′
